# Prediction of Abrasive Waterjet Machining Parameters of Military-Grade Armor Steel by Semi-Empirical and Regression Models

**DOI:** 10.3390/ma15124368

**Published:** 2022-06-20

**Authors:** Soundarapandian Rammohan, Sundaresan Thirumalai Kumaran, Marimuthu Uthayakumar, Kinga Korniejenko, Marek Nykiel, Arumugam Velayutham

**Affiliations:** 1 Department of Automobile Engineering, Kalasalingam Academy of Research and Education, Krishnankoil 626126, Tamil Nadu, India; rammohanmfg@gmail.com; 2 Faculty of Mechanical Engineering, Kalasalingam Academy of Research and Education, Krishnankoil 626126, Tamil Nadu, India; m.uthayakumar@klu.ac.in; 3 Faculty of Materials Engineering and Physics, Cracow University of Technology, al. Jana Pawła II 37, 31-864 Kraków, Poland; kkorniejenko@pk.edu.pl (K.K.); marek.nykiel@pk.edu.pl (M.N.); 4 Former Scientist ‘G’, Combat Vehicles Research and Development Establishment, Ministry of Defense, Chennai 600054, Tamil Nadu, India; vel_sivam@yahoo.com

**Keywords:** abrasive waterjet machining, armor steel, Buckingham π-theorem, regression model

## Abstract

Rolled homogeneous armor steel (RHA) with a high tensile strength, toughness, and hardness is often used in military combat vehicles. RHA is a high-strength low alloy steel suitable for all battlefield usage in military vehicles. The present work examines the prediction output responses in the material removal rate (MRR), surface roughness (Ra), and kerf angle (Ka) for the AWJM of armor steel using regression and semi-empirical models. The AWJM trials were performed using an L27 factorial design with each process variable set to three levels, namely, the standoff distance (SOD), jet traversing speed (JT), and jet water pressure (P). A regression model was constructed using the response surface method (RSM) and data from the trials. Through dimensional analysis and with Buckingham’s π-theorem, a semi-empirical model was built using both the experimental data and material property data. Predictions made by the models were proportionate with the results of the experiments under the same conditions. Microscopic investigations on MRR and Ra were performed using a scanning electron microscope (SEM). The optimal values of the output responses of the machined armor steel plate were obtained with higher MRR = 298.92 mm^3^/min, lower Ka = 0.651°, and lower Ra = 2.23 µm. The present work established that semi-empirical models accurately predict the output responses in the AWJM of armor steel.

## 1. Introduction

Rolled homogeneous armor (RHA) is often utilized in many armor applications [1,2]. Because of its great energy-absorbing qualities, RHA steel is primarily utilized to build military equipment such as tanks, howitzers, and other armored combat vehicles [3,4]. RHA steel is used for ballistic protection in military and civil applications because of its high hardness and ultimate tensile strength. The ability to enhance its hardness with good toughness has been critical to its effectiveness in achieving high performance. This steel has maintained its high position due to its low price, dependability, access to manufacturing infrastructure, continuous value such as structural material, and simplicity of fabrication [5,6]. Due to their high hardness and strength, these steels cannot be turned or milled. This research attempted to cut RHA steel using an unconventional machining method called abrasive waterjet machining (AWJM) [6,7]. Due to the constraint of traditional methods, non-conventional methods are used for material removal. AWJM is an emerging non-conventional process for hard materials such as RHA steels. This is particularly due to characteristics such as the absence of a heat-affected zone, a high-cutting process, the number of co-proficiencies, and the ability to cut complex shapes, among others. Water and a flow of abrasives arrive from two directions in AWJM and become mixed up, and then flow through the nozzle. The impelling force of water is transferred to abrasives. Consequently, the velocity of the abrasives is swiftly increased, and they become efficient in cutting any material. The metal removal process is divided into two zones, referred to as the cutting wear zone and the deformation wear zone [8,9]. The material removal in AWJM includes erosion, microchip development, ploughing, and rubbing [10].

The abrasive striking angle and mechanical characteristics of the target material have been critical to the development of each of these mechanisms. AWJM is gaining acceptance as a standard cutting tool in industries such as nuclear, oil, foundry, automotive, aerospace, and construction. This method may be used in conjunction with wire electric discharge machining, plasma flame, and laser cutting. Compared to plasma and laser machining, the cost of AWJM is typically one-quarter that of wire EDM, and it can cut thicker plates with superior dimensional precision [11,12]. Uthayakumar et al. [13] identified that the performance of the AWJM of the nickel-based superalloy was determined using three machining parameters such as jet pressure (P), jet traverse speed (JT), and standoff distance (SOD). Chen et al. [14] developed a semi-empirical depth equation to optimize the AWJM of the ceramic material. Lemma et al. [15] explained the nozzle oscillation method that was used to investigate the issue of striation on mild steel and aluminum. Mustafa et al. [16] proved that AWJM was used to investigate six different JT in an Inconel 718 nickel-based superalloy. They concluded that as the JT increased, the Ra and the kerf taper increased with decreasing taper width. Kumar et al. [17] explained that the AWJM was used to study the tungsten-aluminum composites. A scanning electron microscope (SEM) measured the sliced surface. They found that JT and SOD had a significant impact on the material removal rate and Ra. Yuvaraj and Kumar [18] showed that AISI D2 steel was examined on the AWJM by adjusting the nozzle impact angle and P to determine the surface quality. They determined that the surface quality was affected by P and the nozzle impact angle. Kumar et al. [19] studied that analysis of variance (ANOVA) and RSM’s Box–Behnken design were used to examine the process variables of the AWJM procedure in depth. The surface quality was determined to depend on JT and the abrasive flow rate. Ishfaq et al. [20], using AWJM, investigated the effect of output factors on the material removal rate (MRR) and kerf taper (Kt) of the clad composite (stainless steel with mild steel). The abrasive flow rate and JT were the most critical factors for the kerf taper and MRR, respectively. Miao et al. [21] investigated the penetration depth in the cutting of (304) stainless steel and (6061) aluminum alloy stacks by using AWJM. Stack components made of the same and various materials and their effects of varying thickness were considered for the studies. The cutting quality improved when the difficult-to-machine material was in the top position.

Wang [22] conducted an experimental model for the cut depth based on the Buckingham theorem and dimensional analysis. On alumina ceramics, tests were conducted in a multipass AWJ to determine the effect of various parameters on the depth of cut. Yuan et al. [23] investigated the grinding of circular pockets on a titanium alloy using AWJM. The Box-Behnken approach examines the input and output parameters, and it has developed a dimensional analysis to compare the experimental and empirical models for milling depth. The average milling depth variations between experimental and forecasted values was 3.5%. Shanmugam and Masood [24] created a semi-empirical model for predicting the kerf taper and verified it using an average percentage deviation and standard deviation. Singh and Shukla [25] studied AWJM, where semi-empirical dimensional analysis models had been developed and effectively used to estimate the kerf angle and Ra, respectively. Pahuja and Ramulu [26] created a semi-empirical model to determine the depth of cut in the AWJM of Ti6Al4V, CFRP, and layered Ti/CFR. Mohankumar et al. [27,28,29] developed using AWJM semi-empirical dimensional analysis-based methods to determine the cutting depth, the angle of the kerf, and the roughness of the surface on MMC.

Profile cutting in armor steels is now conducted by fame-cutting. This method generates a large heat affect zone (HAZ) and poor surface quality, which require yet another operation. However, AWJM overcomes these drawbacks and produces minimal distortion in the work material. AWJM provides the following benefits over other conventional machining processes: no heat deformation; good cutting flexibility; and good surface quality. The suggested work may be developed further using this as a starting point. In this study, Buckingham’s π-theorem–based dimensional analysis was used to create a semiempirical model for predicting the process parameters to identify the effect of the output response. The L27 Taguchi orthogonal array was used to measure the process parameters during these AWJM trials. ANOVA and regression modeling were used to determine the influence of the process parameters. The effect of the process parameters was investigated using a regression model fitted using RSM and ANOVA. Microscopic analysis was used to determine the surface quality under varying process parameters.

## 2. Experimental Setup

**Figure 1:**
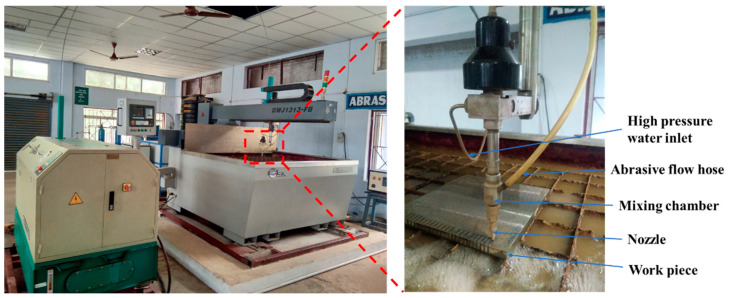
The experimental setup and close-up view of a workpiece mounted on the machine.

The experimental setup used in this work is shown in Figure 1.

AWJM trials were carried out on armor steel using M/s. DADRI International makes the DWJ FB-1313 machining center. Throughout the cutting process, the parameters of jet impact angle (90°), garnet abrasive size (80 mesh), nozzle diameter (0.67 mm), and orifice diameter (0.25 mm) were maintained at constant. On the basis of the literature and initial experiments, the AWJM input parameters and their ranges were chosen. AWJM was used to cut a 20 mm straight linear single pass on the armor steel plate. The process parameters were varied at three levels: [P = 220, 240, 260 MPa; JT = 5, 10, 15 mm/min; and SOD = 1, 2, 3 mm]. The full factorial design was considered to conduct the experiments.

The armor steel material is mainly used in battlefield tank body structures and various combat applications in the defense industry. The chemical composition of armor steel consists of various elements (i.e., C, 0.3301%; Mn, 0.563%; Si, 0.1937%; S, 0.0019%; Cr, 1.507%; Ni, 1.570%; Mo, 0.3857%; V, 0.119%; Al, 0.0494; and Fe, balance). Work on developing semi-empirical models for considering responses was expanded in this study. In this study, four quality requirements were chosen that significantly impact the machining of the material using the AWJM technique. The MRR for abrasive waterjet cutting depends on the penetration depth, kerf width, and JT. It was also observed that the MRR was affected only by the abrasive phase’s power, and not by the water phase. Equation (1) was used to find out the MRR. The kerf characteristics were measured using ImageJ software to find the top kerf widths (K_tw_) and bottom kerf widths (K_bw_). Equation (2) was used to calculate the performance parameter kerf angle (Ka).

(1) $$\mathrm{MRR}=h_{t}d_{i}JT$$

where $h_{t}$ = depth of penetration, $d_{i}$ = kerf width [(K_tw_ + K_bw_)/2], K_tw_ = top kerf width, K_bw_ = bottom kerf width.

(2) $$\mathrm{Kerf}\;\mathrm{angle}=\tan^{-1\;}\left[K_{\mathrm{tw}}-K_{\mathrm{bw}}\right]$$

Surface roughness was measured using a Mitutoyo Corporation, Japan, SJ–401 surface tester M/s, a conical stylus with a tip radius of 5 µm, a probe measuring range of 350 µm, and a speed of 0.25 m/s. When calculating the average Ra in three regions, the sample measurement length was kept constant at 5 mm (top, middle, and bottom).

## 3. Semi-Empirical and Statistical Model

### 3.1. Buckingham’s “π”-Theorem

Dimensional analysis is a technique used to gather a specific set of data on a certain occurrence. A full set of dimensionless parameters may be obtained after the dimensional analysis. The dimensional analysis method is mostly used to make a physical problem easier to solve by cutting down on the number of factors. This may not significantly affect the current problem. Buckingham’s π-theorem can integrate all parameters in a given issue into a dimensionless product ($\pi_{n}$). Algebraic expressions, called terms, are used to produce the necessary mathematical expression related to all the variables in the problem. In this section, a predictive model was established to calculate the performance characteristics including the MRR, Ra, K_tw_, and kerf angle. These models were constructed using dimensional analysis and were based on appropriate process parameters such as P, JT, and SOD in the AWJM process. The performance characteristics evaluated in this study are expected to vary depending on the machining parameters used and the material and thermal properties of the workpiece. Table 1 lists the parameters used in the development of the semi-empirical models and their dimension formulas.

### 3.2. RSM Modeling

The regression analysis method includes experiments, mathematical tools, and statistical analysis. The present investigation’s experimental results given in Table 2 are thoroughly examined. The output response variables MRR, RA, and Ka, are predicted using MINITAB 18 software utilizing a multilinear stepwise regression analysis. The regression model is written as follows with a sample size of n observations for the dependent variable Y (output response) [16,29]:

(3) $$Y=\mathrm{Ao}+\;\sum_{a=1}^{\infty }A_{a}X_{a}+\sum_{a=1}^{\infty }A_{\mathrm{aa}}{\;X}_{a}^{2}+\sum_{a=1}^{\infty }=\sum_{b>1}^{\infty }A_{\mathrm{ab}}X_{a}X_{b}+\varepsilon$$

where Y = output response (Ka, MRR, Ra); Ao = co-efficient; A_aa_ = quadratic co-efficient; A_ab_ = interaction co-efficient; $A_{a},A_{b}$ = dimensionless coded independent variables. The output responses may be described as a function of the AWJ machining parameters such as (JT), (P), and (SOD) through a second-order polynomial regression equation. Equations (4)–(6) can be reformulated as follows to find a connection between the AWJ process parameters and the output responses:

MRR = 1039 + 38.0 SOD − 19.8 JT − 7.9 P − 5.69 SOD × SOD + 0.285 JT × JT + 0.0154 P × P+ 0.91 SOD × JT − 0.074 SOD × P + 0.1027 JT × P(4)

Ra = 25.8 + 0.46 SOD − 0.198 JT − 0.189 P − 0.616 SOD × SOD + 0.00635 JT × JT + 0.000348 P × P − 0.0167 SOD × JT + 0.00893 SOD × P + 0.00074 JT × P(5)

Ka = 2.87 + 0.091 SOD − 0.0203 JT − 0.0187 P + 0.0084 SOD × SOD+ 0.000554 JT × JT + 0.000041 P × P − 0.00036 SOD × JT − 0.000342 SOD × P + 0.000028 JT × P(6)

## 4. Results and Discussion

### 4.1. Experimental and Modeling of MRR

The machining process parameters and the physical and thermal characteristics of the workpiece material were taken into account while creating the models. According to Equation (7), the MRR performance was calculated. The dimensional analysis was used to determine the relationship between the performance measure MRR and the parameters.

MRR = f (JT, SOD, P, AF, ρ, C_p_, K, MT, H_v_) (7)

The “n” variables are represented as (n − p) non-dimensional groups of the variables, referred to as “π” groups using this approach. This dimensional analysis method is based on Buckingham’s π-theorem. Mass (M), length (L), time (T), and temperature (θ) are the primary dimensions in this present study. As a consequence, since there are 10 variables and four fundamental dimensions, n = 10 and r = 4, therefore, n − r = 6 according to the present study. Therefore:

f (π_1_, π_2_, π_3_, π_4_, π_5_, π_6_) = 0 (8)

Dimensional analysis was used to find the six terms and Equation (8) may be rewritten as Equation (9). The details of the calculated terms are as follows:

(9) $$\pi_{1}\;=\;\alpha \;({\pi_{2}}^{a},\;{\pi_{3}}^{b},\;{\pi_{4}}^{c},\;{\pi_{5}}^{d},\;{\pi_{6}}^{e})\;\pi_{1}\;=\;{\left[\mathrm{JT}\right]}^{a1}\;{\left[\mathrm{SOD}\right]}^{b1}\;{\left[P\right]}^{c1}\;{\left[\mathrm{MT}\right]}^{d1}\;\mathrm{MRR},\;M^{0}L^{0}T^{0}\theta^{0}\;=\;{\left[{\mathrm{LT}}^{-1}\right]}^{a_{1}}\;{\left[L\right]}^{b_{1}}\;{\left[{\mathrm{ML}}^{-1}T^{-2}\right]}^{C_{1}}\;{\left[\theta \right]}^{d_{1}}{\;L}^{3}T^{-1}$$

By replacing a_1_, b_1_, c_1_, d_1_ with a, b, c, d; rearranging and rewriting the above phrase as a consequence, the following expression is generated.

$$M^{0}L^{0}T^{0}\theta^{0}=[M{]}^{+c}[\;L{]}^{\;a+b-c+3}\;{\left[T\right]}^{-a-2c-1}\;{\left[\theta \right]}^{d}$$

According to the power indices on both sides, the dimensionless coefficients are shown in the following way:

For θ→0, d=0For M→0, c=0For T→0,−a−2c−1=0∴ a−1=0∴ a=−1For L→0, a+b−c+3∴ b+2=0∴ b=−2

Thus, Equation (10) is developed by replacing the calculated value of the power index of the $\pi_{1}$ term.

(10) $$\pi_{1}=\frac{\mathrm{MRR}}{\left(\mathrm{JT}\right){\left(\mathrm{SOD}\right)}^{2}}$$

Similarly, the values of the following π terms are calculated using the same techniques as for the first term. Table 3 shows the obtained equations. The equation derived by substituting all of the acquired π terms in Equation (9) is provided in Equation (10).

(11) $$\frac{\mathrm{MRR}}{\left(\mathrm{JT}\right)\cdot \left({50D}^{2}\right)}={\left[\frac{\mathrm{JT}\cdot \mathrm{AF}}{\left({\mathrm{SOD}}^{2}\right)\cdot P}\right]}^{a}{\left[\frac{\left({\mathrm{JT}}^{2}\right)\cdot \rho }{P}\right]}^{b}{\left[\frac{\left({\mathrm{JT}}^{2}\right)\cdot \left(\mathrm{MT}\right)\left(C_{P}\right)}{\left({\mathrm{SOD}}^{4}\right)}\right]}^{C}{\left[\frac{\left(\mathrm{MT}\right)\cdot K}{\left(\mathrm{JT}\right)\cdot \left(\mathrm{SOD}\right)\cdot P}\right]}^{d}{\left[\frac{\left({\mathrm{JT}}^{2}\right)\cdot L}{\left({\mathrm{SOD}}^{4}\right)}\right]}^{e}$$

Equation (8) is given as Equation (6) by rearranging the terms:

(12) $$\mathrm{MRR}=\alpha \;\mathrm{JT}\cdot \;{\mathrm{SOD}}^{2}{\left[\frac{\mathrm{JT}\cdot \mathrm{AF}}{\left({\mathrm{SOD}}^{2}\right)\cdot P}\right]}^{a}{\left[\frac{\left({\mathrm{JT}}^{2}\right)\cdot \rho }{P}\right]}^{b}{\left[\frac{\left({\mathrm{JT}}^{2}\right)\cdot \left(\mathrm{MT}\right)\left(C_{P}\right)}{\left({\mathrm{SOD}}^{4}\right)}\right]}^{C}{\left[\frac{\left(\mathrm{MT}\right)\cdot K}{\left(\mathrm{JT}\right)\cdot \left(\mathrm{SOD}\right)\cdot P}\right]}^{d}{\left[\frac{\left({\mathrm{JT}}^{2}\right)\cdot L}{\left({\mathrm{SOD}}^{4}\right)}\right]}^{e}$$

The machining process parameters and the thermal-physical characteristics of the work material are considered in the semi-empirical MRR model, as stated in Equation (12). In Equation (12), the coefficients (α) and the power indices (a, b, c, d, and e) must be determined. Therefore, an experiment was carried out. The coefficients and power indices of Equation (12) were calculated using the nonlinear estimation.

From the ANOVA results in Table 4, it is evident that Jt 85.38% had the greatest effect on MRR compared to the other two process parameters. At A × B 0.31%, A × 0.03%, and B × C 1.56%, the influence of the interaction terms was insignificant (0.31%, 0.03%, and 1.56%, respectively). The impacts of all of the process parameters were obtained by maintaining the performance parameter, one variable constant which is kept constant.

**Figure 2:**
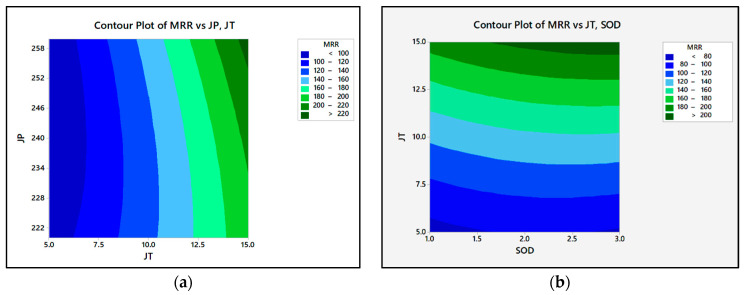
The contour plots of the MRR: (**a**) MRR vs. P, JT; (**b**) MRR vs. JT, SOD.

**Figure 3:**
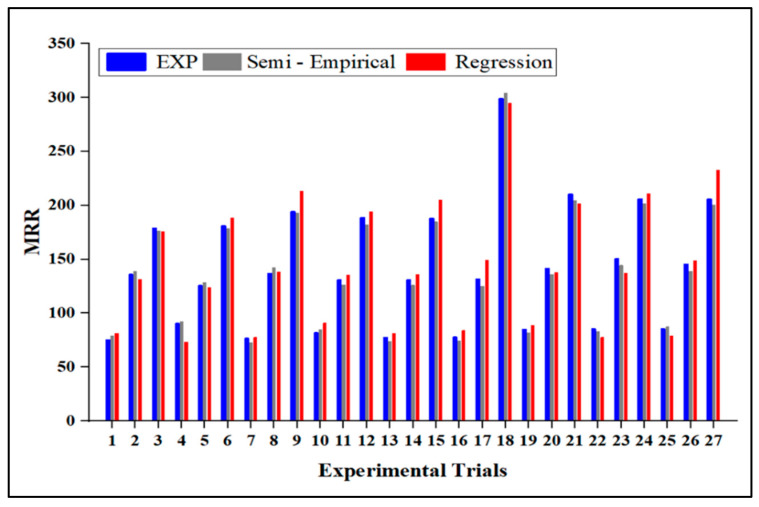
The MRR comparison between the experimental, semi-empirical, and regression methods.

Figure 2 illustrates the contour plots of the MRR. It is the most influencing parameter in the MRR compared to P and SOD. It was observed that when JT increased, the MRR increased. Abrasive waterjet particles in the waterjet stream erode high-strength materials such as armor steel, resulting in material loss. Thus, increased kinetic energy increases the erosion rates and material removal rates. The machining rate increases with a higher traverse speed, removing more material from the workpiece. In turn, the material removal rate is mainly determined by the speed of the traverse, which is consistent with previous research [30,31]. The semi-empirical modeling findings were compared with the experimental data and a regression model. Figure 3 illustrates the trends for semi-empirical modeling, the experimental, and regression model results for each observation number. In the semi-empirical model, the findings demonstrate a good agreement between the experimental and predicted values. The semi-empirical modeling was also more accurate and precise than the regression model. The percentage error variance between the experimental and predicted values was less than 5%. The MRR range changes depending on the JT. With increasing JT and P, the MRR value rises.

**Figure 4:**
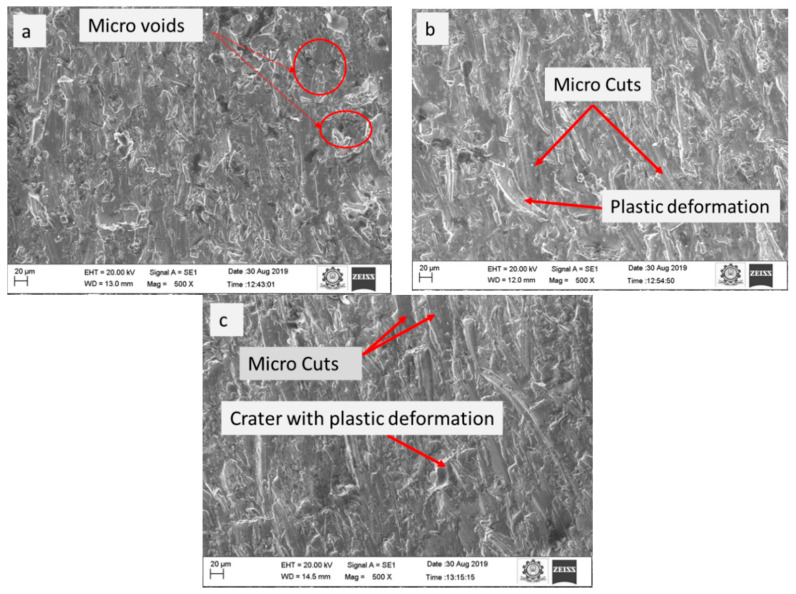
The surface morphology of the machined samples: (**a**) 5 mm/min, (**b**) 10 mm/min, and (**c**) 15 mm/min.

The SEM image of the materials machined at a JT of 5, 10, and 15 mm/min is shown in Figure 4. Figure 4a illustrates the JT at a speed of 5 mm/min. At this speed, more micro voids are detected. The micro-cut and plastic deformation were observed at a JT of 10 mm/min, as illustrated in Figure 4b. Few micro gaps were found at 15 mm/min than at 5 mm/min in Figure 4c. The present study showed that a higher MRR 298.92 mm^3^/min was achieved by increasing the JT to 15 mm/min, with a SOD = 2 mm and P = 260 MPa.

### 4.2. Experimental and Modeling of Ra

The modeling of Ra was determined in a similar manner to that of MRR. The π_1_ term varies and is generated by semi-empirical modeling. The performance parameter Ra is expressed as:

Ra = f (JT, SOD, P, AF, ρ, C_p_, K, MT, H_v_) (13)

As in MRR, the solution for Ra is found in the form of a product of six independent dimensionless (π_n_) terms, as provided in Equation (11).

f (π_1_, π_2_, π_3_, π_4_, π_5_, π_6_) = 0 (14)

Dimensional analysis is used to find the six terms and Equation (14) is redefined as in Equation (15).

(15) $$\pi_{1}=\beta \;({\pi_{2}}^{a},\;{\pi_{3}}^{b},\;{\pi_{4}}^{c},\;{\pi_{5}}^{d},\;{\pi_{6}}^{e})$$

The terms in Equation (12) are rewritten to calculate the π_1_ term as follows:

π_1_ = [JT]^a1^ [SOD]^b1^ [P]^c1^ [MT]^d1^ Ra

The power indices were calculated in the same manner as explained in Section 4.1 for the MRR modeling formulation. The dimensionless coefficients (a, b, c, and d) had values of {0, 1, 0, and 0}. Thus, by replacing the derived values of the power indices for the Ra model, the produced π1 term is provided in Equation (16).

π_1_ = [JT]^0^ [SOD]^1^ [P]^0^ [MT]^0^ Ra (16)

(17) $$\pi_{1}=\mathrm{SOD}\cdot \;\mathrm{Ra}$$

Using Equation (17), the formula for Ra is produced by replacing all the derived terms. The remaining terms (i.e., $\pi_{2},\pi_{3},\pi_{4},\pi_{5},\pi_{6}$) will remain constant as given in the semi-empirical modeling of the MRR Equation (11).

(18) $$\mathrm{SOD}\;\mathrm{Ra}={\left[\frac{\mathrm{JT}\cdot \mathrm{MF}}{\left({\mathrm{SOD}}^{2}\right)\cdot P}\right]}^{a}{\left[\frac{\left({\mathrm{JT}}^{2}\right)\cdot \rho }{P}\right]}^{b}{\left[\frac{\left({\mathrm{JT}}^{2}\right)\cdot \left(\mathrm{MT}\right)\left(C_{P}\right)}{\left({\mathrm{SOD}}^{4}\right)}\right]}^{C}{\left[\frac{\left(\mathrm{MT}\right)\cdot K}{\left(\mathrm{JT}\right)\cdot \left(\mathrm{SOD}\right)\cdot P}\right]}^{d}{\left[\frac{\left({\mathrm{JT}}^{2}\right)\cdot L}{\left({\mathrm{SOD}}^{4}\right)}\right]}^{e}$$

Rearranging the terms and rewriting Equation (14) is provided in Equation (15).

(19) $$\mathrm{Ra}=\frac{\beta }{50D}{\left[\frac{JT\cdot MF}{\left(SOD^{2}\right)\cdot P}\right]}^{a}{\left[\frac{\left(JT^{2}\right)\cdot \rho }{P}\right]}^{b}{\left[\frac{\left(JT^{2}\right)\cdot \left(MT\right)\left(C_{P}\right)}{\left(SOD^{4}\right)}\right]}^{C}{\left[\frac{\left(MT\right)\cdot K}{\left(JT\right)\cdot \left(S0D\right)\cdot P}\right]}^{d}{\left[\frac{\left(JT^{2}\right)\cdot L}{\left(SOD^{4}\right)}\right]}^{e}$$

The power indices β, a, b, c, d, and e, are to be determined. Therefore, an experiment was carried out, and the coefficients and power indices of Equation (19) were calculated using the nonlinear estimation. The ANOVA findings in Table 5 show that JT has the most significant effect on Ra compared to the other two machining factors.

**Figure 5:**
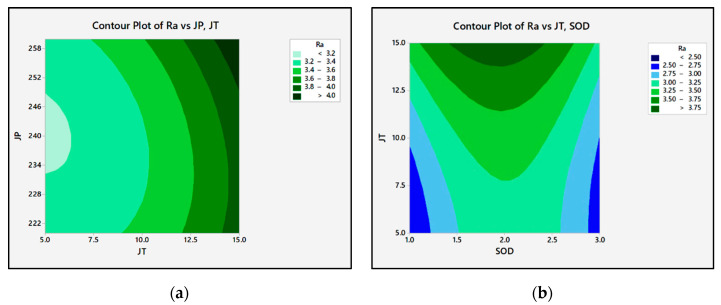
The contour plots of Ra: (**a**) Ra vs P, JT; (**b**) Ra vs. JT, SOD.

Figure 5 shows the contour plot of P, JT, and SOD in Ra. When increasing the SOD from 1 mm to 2 mm, Ra gradually increases from 2.326 µm to 4.516 µm and after that decreases to 2.94 µm at 3 mm. Ra increases from 3.14 µm to 3.28 µm when P is increased from 220 MPa to 260 MPa. The roughness of the surface is directly related to the JT.

From 2.85 to 3.58 µm, the Ra is directly proportional to JT. P increases the particle’s kinetic energy and substantially increases its MRR, while decreasing Ra [32,33].

In this study, Ra is initially low as P increases. But the Ra increases as the P increase further. It is an identity that increasing the P (up to 240 MPa) reduces the Ra. Minimizes the chances of plastic deformation and micro-cutting. Increases in P and JT will alter the roughness of the surface. It is due to the size and type. The P and its interaction do not affect the roughness of the character in this case. When P is increased, abrasive fragments is also increased along with their enhanced rate. Due to fragmentation, the size of impacting abrasive particles is reduced. Similarly, when P is increased, the kinetic energy of the water jet also increased, affecting the Ra. The JT, second-order terms of P, and JT are identified as significant process parameters for Ra, based on the experimental results. The standoff distance does not have any physical effect on the roughness of the surface, as previously stated in the literature [34,35]. With a large SOD, more contact P is produced than with a small SOD. It makes a better machining surface [36,37]. Ra is predicted to be reduced by increasing JT since the number of abrasive particles that are imposed on the workpiece gets reduced by increasing JT.

**Figure 6:**
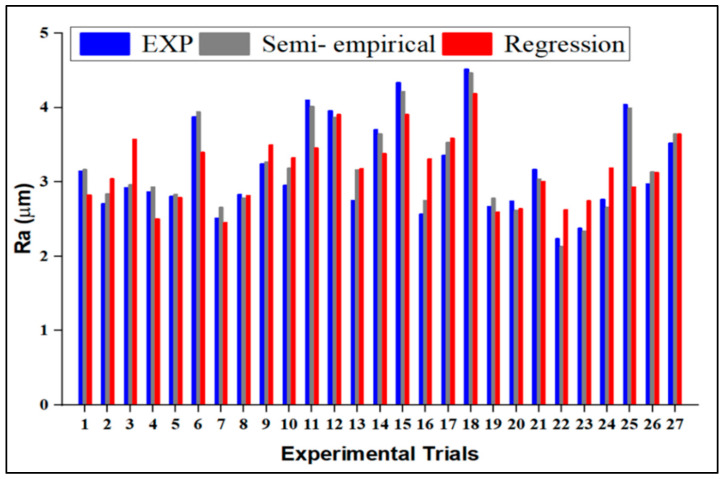
Comparison between experimental, semi-empirical and regression methods.

The semi-empirical modeling is validated by evaluating it to Ra’s experimental data and regression model. The variations for each observation number for semi-empirical, observed, and regression model Ra values are shown in Figure 6. The results from the semi-empirical modeling indicates a strong level of correlation between experimental and predicted values. According to Figure 6, semi-empirical models are more precise than regression models. The difference between the observed and predicted values is less than 5%.

**Figure 7:**
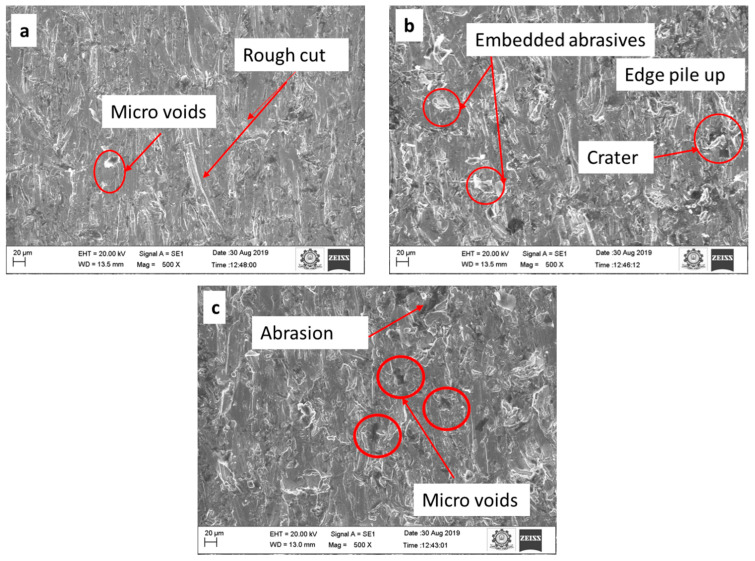
AWJM surface micrograph at SOD = 2 mm, JT = 15 mm/min, and P = 260 MPa (**a**) top, (**b**) middle, and (**c**) bottom region.

The trial no. 22 is chosen because it provides a lower Ra value than the other experiments. Figure 7a–c illustrate the surface quality of the top, middle, and bottom sections.

**Figure 8:**
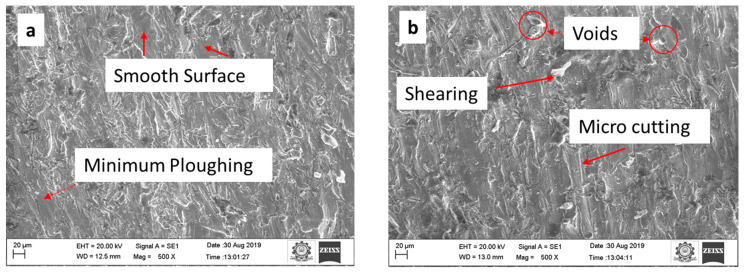

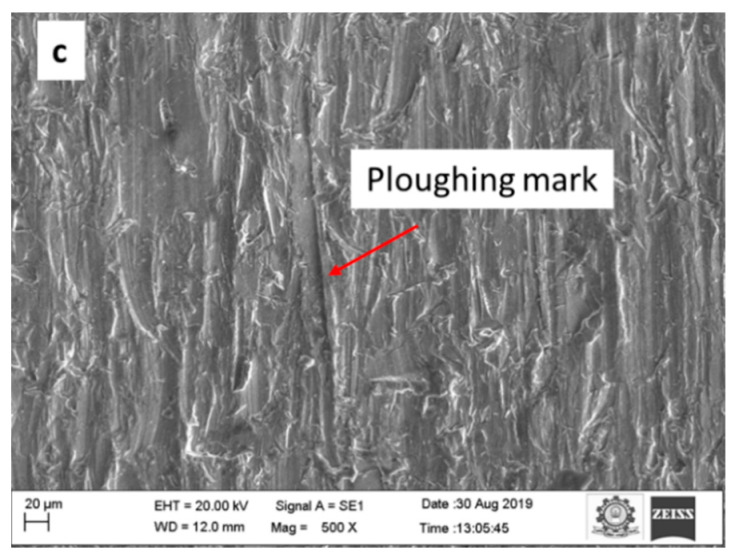
AWJM surface micrograph at SOD = 3 mm, JT = 5 mm/min, and P = 240 MPa (**a**) top, (**b**) middle, and (**c**) bottom region.

At a higher JT (15 mm/min), the machined surface appears with more microvoids, rough cut, edge pile up, and crater, whereas at a lower JT (5 mm/min), the machined surface appears with a full cut and smooth surface, minimal plowing, less void, and less shearing as shown in Figure 8a–c. It is possible to achieve minimum roughness even at low JT because abrasive particles have high kinetic energy and may thus penetrate. Better surface finish is obtained at trial no. 22, with SOD = 3 mm, JT = 5 mm/min and P = 240 MPa.

### 4.3. Experimental and Modelling of K_tw_, K_bw_, and Ka

The models of kerf top width (K_tw_), and kerf bottom width (K_bw_) are determined in this investigation. Ka is calculated using the performance measure of K_bw_ width. The π1 term for K_tw_ and K_bw_ are generated by semi-empirical modelling. K_tw_ can be expressed in the formula provided in Equation (20).

K_tw_ = f (JT, SOD, P, AF, ρ, C_p_, K, MT, H_v_) (20)

The power indices are calculated similarly to the MRR and Ra model formulations. The dimensionless coefficients a, b, c, and d have values of 0, 1, 0, and 0 respectively. Thus, by replacing the acquired power indices values for the K_tw_ width model, the derived π_1_ term is provided in Equation (21).

$$\pi_{1}=\gamma \;({\pi_{2}}^{a},\;{\pi_{3}}^{b},\;{\pi_{4}}^{c},\;{\pi_{5}}^{d},\;{\pi_{6}}^{e})$$

π_1_ = [JT]^a1^ [SOD]^b1^ [P]^c1^ [MT]^d1^ T_k_ (21)

π_1_ = [JT]^0^ [SOD]^1^ [P]^0^ [MT]^0^ T_k_

π_1_ = SOD · K_tw_

The remaining terms $\pi_{2},\pi_{3},\pi_{4},\pi_{5},\pi_{6}$ will stay constant from the semi-empirical modeling of MRR, and the formula for the K_tw_ width produced by replacing all the attained terms is given in Equation (22).

(22) $$K_{\mathrm{tw}}=\frac{\gamma }{50D}{\left[\frac{\mathrm{JT}\cdot \mathrm{MF}}{\left(SOD^{2}\right)\cdot P}\right]}^{a}{\left[\frac{\left({\mathrm{JT}}^{2}\right)\cdot \rho }{P}\right]}^{b}{\left[\frac{\left({\mathrm{JT}}^{2}\right)\cdot \left(\mathrm{MT}\right)\left(C_{P}\right)}{\left(SOD^{4}\right)}\right]}^{C}{\left[\frac{\left(\mathrm{MT}\right)\cdot K}{\left(\mathrm{JT}\right)\cdot \left(SOD\right)\cdot P}\right]}^{d}{\left[\frac{\left({\mathrm{JT}}^{2}\right)\cdot L}{\left(SOD^{4}\right)}\right]}^{e}$$

where, γ is the proportionality constant in Equation (22)

The values of the power indices (a, b, c, d, and e) must be determined. Therefore, an experiment is carried out, and the coefficients and power indices of Equation (18) have been calculated using the nonlinear estimation, respectively.

The K_bw_ must be determined to calculate the Ka model. The performance parameter Ka depends on both K_tw_ and K_bw_, which necessitates a semi-empirical model usage for K_bw_. The experiment was carried out to determine the values of K_tw_ and K_bw_. The width of K_bw_ is directly proportional to the width of the K_tw_. As a result, the $\pi_{1}$ term of K_tw_ corresponds to the $\pi_{1}$ term of K_bw_ as provided in Equation (23).

(23) $$\pi_{1}=\mathrm{SOD}\cdot \;K_{\mathrm{bw}}$$

(24) $$K_{\mathrm{bw}}=\frac{\delta }{50D}{\left[\frac{JT\cdot MF}{\left(SOD^{2}\right)\cdot P}\right]}^{a}{\left[\frac{\left(JT^{2}\right)\cdot \rho }{P}\right]}^{b}{\left[\frac{\left(JT^{2}\right)\cdot \left(MT\right)\left(C_{P}\right)}{\left(SOD^{4}\right)}\right]}^{C}{\left[\frac{\left(MT\right)\cdot K}{\left(JT\right)\cdot \left(SOD\right)\cdot P}\right]}^{d}{\left[\frac{\left(JT^{2}\right)\cdot L}{\left(SOD^{4}\right)}\right]}^{e}$$

δ is the proportionality constant in Equation (24).

The power indices values, (δ,  a, b, c, d, and e) must be determined. Therefore, an experiment is carried out, and the coefficients and power indices of Equation (24) have been calculated using the nonlinear estimation. Thus, the Ka performance parameter is predicted using the K_tw_ and K_bw_ models. Equation (25) can be used to calculate Ka.

(25) $$\mathrm{Ka}=tan^{-1}\;\left[\frac{K_{\mathrm{tw}}-K_{\mathrm{bw}}}{2t}\right]$$

Table 6 shows that the interaction of SOD, JT, P, and the second-order interaction of JT and P has a physical significance, with SOD having the highest contribution of 50.78% and the second-order interaction of P having a contribution of 2.92%. It was found that the SOD–JT, P, and JT interaction did not affect the Ka.

**Figure 9:**
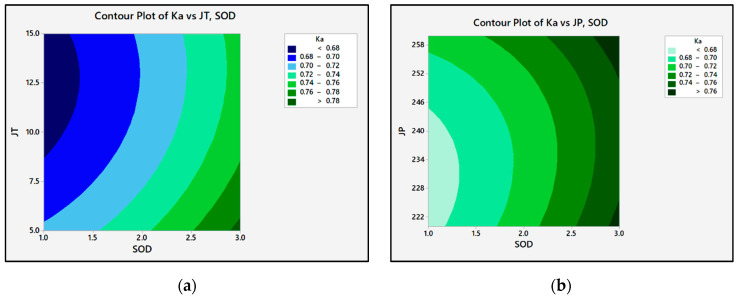
The contour plots of Ka: (**a**) Ra vs. JT, SOD; (**b**) Ka vs. P, SOD.

Figure 9 shows the contour plot of the machining parameters on Ka, which increased linearly with SOD. Ka initially decreased from 0.727 to 0.720 while the P varied. Then, Ka increased exponentially to 0.746 for a further increase in P. There was a significant relationship between SOD, P, JT, and Ka. While the JT increased, the Ka decreased, as shown in Figure 8a. When the JT increased from 5 mm/min to 10 mm/min, the Ka increased from 0.752° to 0.722°; when the JT increased to 15 mm/min, the taper angle was reduced to 0.719°. The variance in Ka was due to the hardness in the material. The SOD and JT should be kept to a minimum. The Ka decreased as the hydraulic P increased. This is because kinetic energy is enlarged in the velocity transfer of abrasives, resulting in a diminished K_bw_. The kinetic energy and cutting performance of the particles were enhanced with increased P. As P increased from 240 MPa to 260 MPa, the top kerf increased due to the increase in Ka. At the base of the workpiece, the waterjet kinetic energy decreased. It could not remove the material efficiently and therefore created a narrow K_bw_, resulting in a decrease in Ka. A similar trend was seen in armor steel cutting. Ka increased with an increase in SOD. At high speeds, the jet will deviate. The jet increased as it exited the nozzle. The workpiece surface is now exposed to the bottom of the waterjet after the SOD is raised.

**Figure 10:**
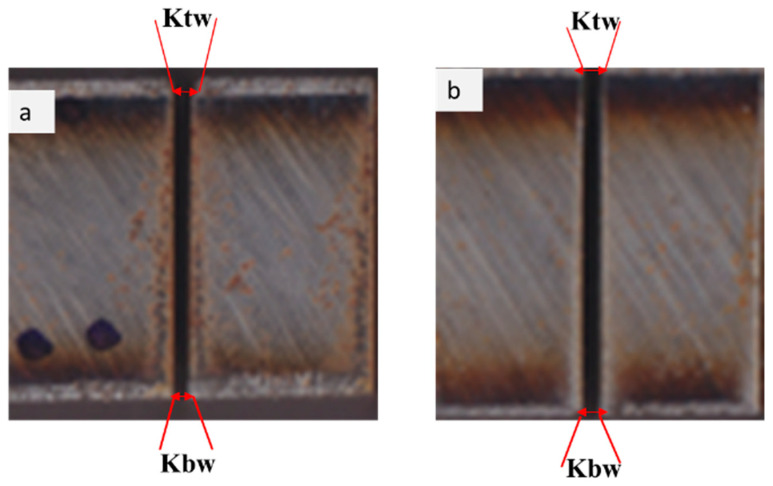
The kerf angle formation at different matching conditions: (**a**) SOD = 1 mm, JT = 10 mm/min, P = 220 MPa, and Ka = 0.651°, (**b**) SOD = 3 mm, JT = 5 mm/min, P = 240 MPa, and Ka = 0.804°.

As the water jet diverged, it lost uniformity, which diminished the cutting area seen in Ka immediately after that. Similarly, increasing JT tended to increase Ka. When the JT value is low, the nozzle allows for a high volume of abrasive particles to strike the workpiece. As a result, a larger volume of material is removed, resulting in a higher K_tw_. At the same time, the waterjet thoroughly penetrates the workpiece, maintaining kinetic energy and efficiently cutting the bottom material. The kerf width variation reduces the taper angle. The correct taper was obtained at SOD (1 mm), JT (10 mm/min), and P (220 MPa). The reverse taper was obtained at SOD (3 mm), JT (5 mm/min), and P (240 MPa), as shown in Figure 10.

**Figure 11:**
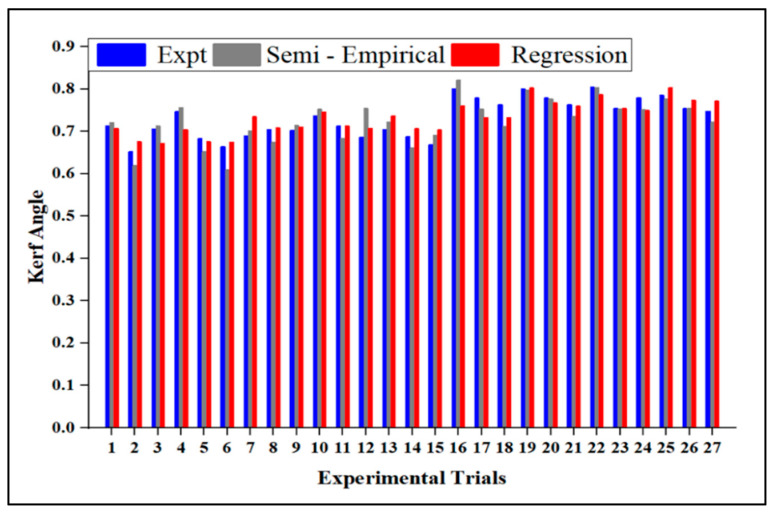
A comparison of Ka between the experimental, semi-empirical, and regression methods.

The Ka semi-empirical model was verified by comparing the experimental data and regression model. Figure 11 shows the variation for each observation number in the semi-empirical modeling, experimental, and regression models with Ka values. The findings of the semi-empirical model showed a good connection between the experimental and predicted values. The semi-empirical models outperformed the regression models. The observed and anticipated values varied by less than 5%.

## 5. Conclusions

The influence of the AWJM process parameters on the MRR, RA, and Ka values while machining an armor steel plate was investigated in this study. Using Buckingham’s π-theorem and the regression equation, a semi-empirical model was created to estimate three key parameters: MRR, Ra, and Ka. ANOVA was used to evaluate the model’s significance and performance. The experimental findings were compared with the predicted values of MRR, Ra, and Ka using the proposed semi-empirical model and regression model, and the following decisions were made:

- The confirmation experiments showed that the residuals between the predicted and experimental values were less than 5%. The values of the semi-empirical modeling and regression models were compared with the experimental values. For MRR, Ra, and Ka, the semi-empirical model was proven to be more accurate than the regression model to predict the relationships of the variable.
- The use of the analysis of variance approach in combination with the dimensional analysis reduced the number of insignificant variables and resulted in simpler models for MRR, Ra, and Ka.
- The maximum MRR was obtained at SOD = 2 mm, JT = 15 mm/min, and P = 260 MPa. Improved MRR was achieved due to the greater kinetic energy of the jet produced by this combination. These findings demonstrate that a higher surface finish was attained with a higher SOD = 3 mm and lower JT = 5 mm/min. This allows for a more uniform MRR, which resulted in a lower roughness. Ra was determined to be 2.23 µm.
- The SOD process parameter significantly impacts the response, Ka. The variation in Ka is due to the hardness of the material. The SOD and JT should be kept at the minimum. The lower Ka (0.651°) was obtained at SOD = 1 mm, JT = 10 mm/min, and P = 220 MPa.
- The microstructural analysis of the machined surfaces revealed three regions: top, middle, and bottom. In the top region, smooth surfaces were obtained, and in the middle region, fewer voids were present, and less ploughing was identified at the bottom.
- In future, the research may be extended on the comparison of experimental results with numerical modeling studies. The results obtained can be applied in battlefield vehicle applications.

## Figures and Tables

**Table 1:** The dimensions of the AWJM process parameters.

Characteristics	Parameters	Symbol	Unit	Dimensions
Output Parameters	Material removal rate	MRR	mm^3^/min	L^3^T^−1^
	Surface roughness	R_a_	µm	L^1^
	Kerf top width	K_tw_	mm	L^1^
	Kerf bottom width	K_bw_	mm	L^1^
Input parameters	Jet traverse speed	JT	mm/min	LT^−1^
	Pressure	P	MPa	ML^−1^T^−2^
	Standoff distance	SOD	mm	L^1^
Material and Thermal Properties	Abrasive Flow rate	AF	g/sec	MT^−1^
	Density	ρ	Kg/m^3^	ML^−3^
	Specific heat capacity	C_p_	J/Kg °C	L^2^T^2^ $\theta^{-1}$
	Thermal conductivity	K	W/mK	MLT^−3^ $\theta^{-1}$
	Melting point	MT	°C	θ
	Heat of vaporization	H_v_	Cal/g	L^2^T^2^

**Table 2:** The full factorial design and experimental results.

Trial No.	SOD (mm)	JT (mm/min)	P (MPa)	MRR (mm^3^/min)	Ra (µm)	K_tw_ (mm)	K_bw_ (mm)	Ka (θ)
1	1	5	220	75.04	3.14	0.895	0.981	0.713
2	1	10	220	135.6	2.71	0.785	0.705	0.652
3	1	15	220	178.8	2.92	0.876	0.819	0.705
4	1	5	240	90.28	2.87	0.964	1.293	0.746
5	1	10	240	125.36	2.80	0.834	0.672	0.683
6	1	15	240	180.72	3.87	0.805	0.762	0.663
7	1	5	260	76.28	2.51	0.857	1.05	0.689
8	1	10	260	136.56	2.83	0.872	0.743	0.704
9	1	15	260	193.8	3.24	0.872	0.835	0.702
10	2	5	220	81.64	2.95	0.938	1.028	0.736
11	2	10	220	130.72	4.10	0.885	0.686	0.712
12	2	15	220	188.52	3.96	0.843	0.791	0.686
13	2	5	240	77.32	2.75	0.881	1.052	0.703
14	2	10	240	130.64	3.70	0.843	0.719	0.687
15	2	15	240	187.44	4.33	0.814	0.819	0.668
16	2	5	260	77.56	2.56	1.062	1.057	0.800
17	2	10	260	131.44	3.36	1.009	0.743	0.778
18	2	15	260	298.92	4.52	0.981	0.786	0.763
19	3	5	220	84.76	2.67	1.062	1.057	0.800
20	3	10	220	141.36	2.74	1.009	0.743	0.778
21	3	15	220	210.24	3.17	0.981	0.786	0.763
22	3	5	240	85.12	2.24	1.071	1.057	0.804
23	3	10	240	150.4	2.37	0.962	0.753	0.754
24	3	15	240	205.8	2.76	1.014	0.866	0.779
25	3	5	260	85.12	4.04	1.033	1.095	0.785
26	3	10	260	145.2	2.97	0.961	0.752	0.753
27	3	15	260	205.56	3.52	0.953	0.862	0.747

**Table 3:** The characteristics of the MRR performance are represented by the π term.

π Term Expression.	The Final Value of π Terms
π_2_ = [JT]^a1^ [SOD]^b1^[P]^c1^ [MT]^d1^ AF	$\frac{\left(\mathrm{JT}\right)\;\left(\mathrm{AF}\right)}{\left({\mathrm{SOD}}^{2}\right)\;\left(P\right)}$
π_3_ = [JT]^a1^ [SOD]^b1^[P]^c1^ [MT]^d1^ ρ	$\frac{\left({\mathrm{JT}}^{2}\right)\cdot \left(\rho \right)}{P}$
π_4_ = [JT]^a1^ [SOD]^b1^[P]^c1^ [MT]^d1^ Cp	$\frac{\left({\mathrm{JT}}^{2}\right)\left(\mathrm{MT}\right)\left(C_{P}\right)}{\left({\mathrm{SOD}}^{4}\right)}$
π_5_ = [JT]^a1^ [SOD]^b1^[P]^c1^ [MT]^d1^ K	$\frac{\left(\mathrm{MT}\right)\cdot K}{\left(\mathrm{JT}\right)\cdot \left(\mathrm{SOD}\right)\cdot P}$
π_6_ = [JT]^a1^ [SOD]^b1^[P]^c1^ [MT]^d1^ H_v_	$\frac{\left({\mathrm{JT}}^{2}\right)\cdot \mathrm{Hv}}{\left({\mathrm{SOD}}^{4}\right)}$

**Table 4:** The ANOVA for MRR.

Source	Sum of Square	DOF	Mean Square	F Value	*p* Value	Contribution, %	Remarks
Model	73,211.5	9	8134.61	17.45	<0.0001	90.23	significant
SOD	815.0	1	815.00	1.75	0.2036	1.00	
JT	69,276.3	1	69,276.35	148.61	<0.0001	85.38	significant
P	850.9	1	850.92	1.83	0.1944	1.05	
AB	250.98	1	250.98	0.5384	0.527	0.31	
AC	26.05	1	26.05	0.0559	0.430	0.03	
BC	1264.85	1	1264.85	2.71	0.1944	1.56	
A^2^	194.18	1	194.18	0.4166	0.4731	0.24	
B^2^	305.12	1	305.12	0.6545	0.8160	0.38	
C^2^	228.00	1	228.00	0.4891	0.1179	0.28	
Error	7924.7	17	466.16			9.77	
Total	81,136.1	26				100	

**Table 5:** ANOVA for Ra.

Source	Sum of Square	DOF	Mean Square	F Value	*p* Value	Contribution, %	Remarks
Model	5.56	9	0.6175	2.29	0.0674	54.81	significant
SOD	0.0091	1	0.0091	0.0336	0.8566	0.09	
JT	2.39	1	2.39	8.88	0.0084	23.61	significant
P	0.0799	1	0.0799	0.2963	0.5933	0.79	
AB	0.0837	1	0.0837	0.3104	0.5847	0.83	
AC	0.3831	1	0.3831	1.42	0.2496	3.78	
BC	0.0656	1	0.0656	0.2432	0.6282	0.65	
A²	2.28	1	2.28	8.44	0.0099	22.44	
B²	0.1512	1	0.1512	0.5608	0.4642	1.49	
C²	0.1160	1	0.1160	0.4304	0.5206	1.14	
Error	4.58	17	0.2696			45.19	
Total	10.14	26				100%	

**Table 6:** The ANOVA for Ka.

Source	Sum of Square	DOF	Mean Square	F Value	*p* Value	Contribution, %	Remarks
Model	0.0383	9	0.0043	4.44	0.0040	70.18	significant
SOD	0.0277	1	0.0277	28.95	<0.0001	50.78	significant
JT	0.0050	1	0.0050	5.21	0.0356	9.14	
P	0.0017	1	0.0017	1.81	0.1960	3.18	
AB	0.0000	1	0.0000	0.0411	0.8418	0.07	
AC	0.0006	1	0.0006	0.5855	0.4547	1.03	
BC	0.0001	1	0.0001	0.0966	0.7597	0.17	
A^2^	0.0004	1	0.0004	0.4449	0.5137	0.78	
B^2^	0.0011	1	0.0011	1.20	0.2887	2.10	
C^2^	0.0016	1	0.0016	1.66	0.2145	2.92	
Error	0.0163	17	0.0010			29.82	
Total	0.0546	26				100	

## References

[1] Bhav Singh B., Sukumar G., Sivakumar K., Madhu V., Arockia Kumar R. (2021). A comparative study on the ballistic performance of high nitrogen steel and rolled homogeneous armour steel against 7.62 mm ball and 12.7 mm armour piercing projectiles. Mater. Today Proc..

[2] Bobbili R., Madhu V., Gogia A.K. (2013). Effect of Wire-EDM Machining Parameters on Surface Roughness and Material Removal Rate of High Strength Armor Steel. Mater. Manuf. Process..

[3] Balakrishnan M., Balasubramanian V., Madhusudhan Reddy G. (2019). Microstructural analysis on ballistic tested armour steel joints fabricated using low hydrogen ferritic consumables for capping pass. Key Eng. Mater..

[4] Hakan Atapek S., Karagoz S. (2011). Ballistic impact behaviour of a tempered bainitic steel against 7.62 mm armour piercing projectile. Def. Sci. J..

[5] Ilhak B., Güner F. (2022). Failure Analysis of Boron Carbide-Reinforced Rolled Homogeneous Armour Against Kinetic Energy Ammunition. Trans. Indian Inst. Met..

[6] Singh B.B., Sukumar G., Senthil P.P., Jena P.K., Reddy P.R.S. (2017). Future Armour Materials and Technologies for Combat Platforms Future Armour Materials and Technologies for Combat Platforms. Def. Sci. J..

[7] Rammohan S., Kumaran S.T., Uthayakumar M., Velayudham A. (2021). Application of TOPSIS Optimization in Abrasive Water Jet Machining of Military Grade Armor Steel. Hum. Factors Mech. Eng. Def. Saf..

[8] Arola D., Ramulu M. (1997). Material removal in abrasive waterjet machining of metals. Surf. Integr. Texture.

[9] Ramakrishnan S. (2022). Investigating the effects of abrasive water jet machining parameters on surface integrity, chemical state in machining of Ti-6Al-4V. Mater. Today Commun..

[10] Hashish M. (1984). A Modeling Study of Metal Cutting with Abrasive Waterjets. J. Eng. Mater. Technol..

[11] Palaniyappan S., Veiravan A., Kaliyamoorthy R., Kumar V. (2022). Sustainable solution to low-cost alternative abrasive from electric ceramic insulator waste for use in abrasive water jet machining. Int. J. Adv. Manuf. Technol..

[12] Kulekci M.K. (2002). Processes and apparatus developments in industrial waterjet applications. Int. J. Mach. Tools Manuf..

[13] Uthayakumar M., Khan M.A., Kumaran S.T., Slota A., Zajac J. (2016). Machinability of Nickel-Based Superalloy by Abrasive Water Jet Machining. Mater. Manuf. Process..

[14] Chen L., Siores E., Wong W.C.K. (1996). Kerf characteristics in abrasive waterjet cutting of ceramic materials. Int. J. Mach. Tools Manuf..

[15] Lemma E., Chen L., Siores E., Wang J. (2002). Optimising the AWJ cutting process of ductile materials using nozzle oscillation technique. Int. J. Mach. Tools Manuf..

[16] Ay M., Çaydaş U., Hasçalik A. (2010). Effect of Traverse Speed on Abrasive Waterjet Machining of Age Hardened Inconel 718 Nickel-Based Superalloy. Mater. Manuf. Process..

[17] Ravi Kumar K., Sreebalaji V.S., Pridhar T. (2018). Characterization and optimization of Abrasive Water Jet Machining parameters of aluminium/tungsten carbide composites. Measurement.

[18] Yuvaraj N., Pradeep Kumar M. (2017). Surface integrity studies on abrasive water jet cutting of AISI D2 steel. Mater. Manuf. Process..

[19] Kumar A., Singh H., Kumar V. (2018). Study the parametric effect of abrasive water jet machining on surface roughness of Inconel 718 using RSM-BBD techniques. Mater. Manuf. Process..

[20] Ishfaq K., Mufti N.A., Ahmed N., Pervaiz S. (2019). Abrasive waterjet cutting of cladded material: Kerf taper and MRR analysis. Mater. Manuf. Process..

[21] Miao X., Wu M., Song L., Ye F., Qiang Z. (2019). Research on the method of stacked cutting of abrasive water jet. Int. J. Adv. Manuf. Technol..

[22] Wang J. (2010). Depth of cut models for multipass abrasive waterjet cutting of alumina ceramics with nozzle oscillation. Front. Mech. Eng. China.

[23] Yuan Y., Chen J., Gao H., Wang X. (2020). An investigation into the abrasive waterjet milling circular pocket on titanium alloy. Int. J. Adv. Manuf. Technol..

[24] Shanmugam D.K., Masood S.H. (2009). An investigation on kerf characteristics in abrasive waterjet cutting of layered composites. J. Mater. Process. Technol..

[25] Singh D., Shukla R.S. (2020). Integration of quality characteristics models as a software-based graphical interface for machining of AA6351 aluminum alloy using abrasive water jet process. J. Braz. Soc. Mech. Sci. Eng..

[26] Pahuja R., Ramulu M. (2019). Abrasive water jet machining of Titanium (Ti6Al_4_V)–CFRP stacks—A semi-analytical modeling approach in the prediction of kerf geometry. J. Manuf. Process..

[27] Mohankumar V., Kanthababu M., Velayudham A. (2021). Abrasive waterjet cutting of boron carbide particles reinforced Al 6063 MMCs—A semi empirical modeling approach in the prediction of kerf angle. Measurement.

[28] Veeraraghavan M., Mani K., Arumugam V. (2022). Prediction of surface roughness using semi-empirical and regression models in machining of metal matrix composites using abrasive waterjet. Int. J. Adv. Manuf. Technol..

[29] Mohankumar V., Kanthababu M. (2020). Semi-empirical model for depth of cut in abrasive waterjet machining of metal matrix composites. J. Braz. Soc. Mech. Sci. Eng..

[30] Çaydaş U., Hasçalik A. (2008). A study on surface roughness in abrasive waterjet machining process using artificial neural networks and regression analysis method. J. Mater. Process. Technol..

[31] Ficko M., Begic-Hajdarevic D., Cohodar Husic M., Berus L., Cekic A., Klancnik S. (2021). Prediction of Surface Roughness of an Abrasive Water Jet Cut Using an Artificial Neural Network. Materials.

[32] Llanto J.M., Tolouei-Rad M., Vafadar A., Aamir M. (2021). Impacts of Traverse Speed and Material Thickness on Abrasive Waterjet Contour Cutting of Austenitic Stainless Steel AISI 304L. Appl. Sci..

[33] Ramulu M., Arola D. (1994). The influence of abrasive waterjet cutting conditions on the surface quality of graphite/epoxy laminates. Int. J. Mach. Tools Manuf..

[34] Arola D., Ramulu M. (1996). A Study of Kerf Characteristics in Abrasive Waterjet Machining of Grahite/Epoxy Composite. J. Eng. Mater. Technol..

[35] Xiong J., Wan L., Qian Y., Sun S., Li D., Wu S. (2022). A new strategy for improving the surface quality of Ti6Al_4_V machined by abrasive water jet: Reverse cutting with variable standoff distances. Int. J. Adv. Manuf. Technol.

[36] Selvam R., Karunamoorthy L., Arunkumar N. (2017). Investigation on performance of abrasive water jet in machining hybrid composites. Mater. Manuf. Process..

[37] Shanmugam A., Krishnamurthy K., Mohanraj T. (2020). Experimental study of surface roughness and taper angle in abrasive water jet machining of 7075 aluminum composite using Response Surface Methodology. Surf. Rev. Lett..

